# Aerosol persistence in relation to possible transmission of SARS-CoV-2

**DOI:** 10.1063/5.0027844

**Published:** 2020-10-01

**Authors:** Scott H. Smith, G. Aernout Somsen, Cees van Rijn, Stefan Kooij, Lia van der Hoek, Reinout A. Bem, Daniel Bonn

**Affiliations:** 1 Van der Waals-Zeeman Institute, Institute of Physics, University of Amsterdam, 1098 XH Amsterdam, The Netherlands; 2 Cardiology Centers of the Netherlands, 1073 TB Amsterdam, The Netherlands; 3 Laboratory of Experimental Virology, Department of Medical Microbiology, Amsterdam UMC, Location AMC, University of Amsterdam, 1105 AZ Amsterdam, The Netherlands; 4 Department of Pediatric Intensive Care, Emma Children’s Hospital, Amsterdam University Medical Centers, Location AMC, 1105 AZ Amsterdam, The Netherlands

## Abstract

Transmission of SARS-CoV-2 leading to COVID-19 occurs through exhaled respiratory droplets from infected humans. Currently, however, there is much controversy over whether respiratory aerosol microdroplets play an important role as a route of transmission. By measuring and modeling the dynamics of exhaled respiratory droplets, we can assess the relative contribution of aerosols to the spreading of SARS-CoV-2. We measure size distribution, total numbers, and volumes of respiratory droplets, including aerosols, by speaking and coughing from healthy subjects. Dynamic modeling of exhaled respiratory droplets allows us to account for aerosol persistence times in confined public spaces. The probability of infection by inhalation of aerosols when breathing in the same space can then be estimated using current estimates of viral load and infectivity of SARS-CoV-2. The current known reproduction numbers show a lower infectivity of SARS-CoV-2 compared to, for instance, measles, which is known to be efficiently transmitted through the air. In line with this, our study of transmission of SARS-CoV-2 suggests that aerosol transmission is a possible but perhaps not a very efficient route, in particular from non-symptomatic or mildly symptomatic individuals that exhibit low viral loads.

## INTRODUCTION

Respiratory droplets form the most important carrier of SARS-CoV-2 virions and may infect humans by direct inhalation or indirectly through hand or object contact. During the current COVID-19 pandemic, numerous explosive local outbreaks, so-called super-spreading events, in public spaces or health care settings have raised concerns of aerosol transmission of SARS-CoV-2. Aerosols, or microdroplets, are formed and exhaled during loud speaking, singing, sneezing, and coughing. As infected persons (initially) may have none or mild symptoms, an aerosol transmission route of SARS-CoV-2 may have tremendous impact on health care strategies to prevent the spreading of COVID-19 in public spaces. Importantly, SARS-CoV-2 viral particles have been detected in microdroplets, which may spread in exhaled air during breathing, talking, singing, sneezing, or coughing by an infected individual.^1–12^

Microdroplets form aerosol clouds, which have a relatively long airborne time,^13^ and may thus pose an important threat to community spread of COVID-19. However, to what extent microdroplets in practice result in infections with the SARS-CoV-2 virus remains a topic of intense debate.^14–21^

Next to virus and host factors, this type of viral transmission through aerosols depends strongly on droplet properties and behavior.^22,23^ In order to aid in the development of effective preventive strategies for SARS-CoV-2 transmission, in this study we measure and model respiratory droplet physics to predict the importance of community SARS-CoV-2 transmission by the aerosol route.

## RESULTS AND DISCUSSION

### Size distribution

**FIG. 1.**
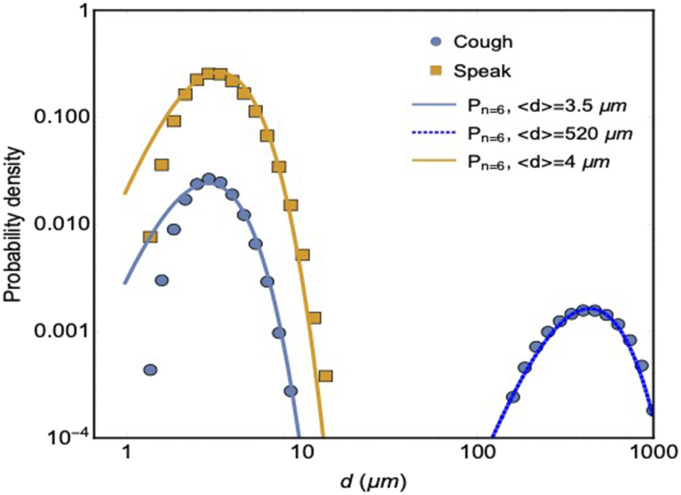
Measured drop size distributions of droplets produced when coughing (circles) and speaking (squares). Solid lines are fits with gamma distributions, where P denotes the probability density and n is a measure for the width of the gamma distribution, see Ref. 23 for details.

We measure size distributions of droplets in aerosols released when speaking or coughing using laser diffraction (Malvern Spraytech®) and consistently find a double-peaked drop size distribution for coughing, and a single-peak drop size distribution for speech, which can be described by a distribution corresponding to a normal liquid spraying process,^23^ as shown in Fig. 1. A previous study^2^ showed that age, sex, weight, and height have no statistically significant effect on the aerosol composition in terms of the size and number of droplets. We tested seven healthy volunteers (five male, two female) and found that the variability in drop production by coughing between the different emitters was relatively small, except for one person, who produced 17 times more liquid volume than the others. It has been suggested that if such a person would be infected with SARS-CoV-2, he or she could become a so-called “super-spreader” due to the high number of droplets emitted.^2,12^

Using a precision balance, the volumes of saliva/mucus produced by the high emitter when coughing or speaking into a small plastic bag were measured by weighing before and after a single cough or saying “Stay Healthy” for ten times.^24^ Averaging over 20 experiments, we find that a single cough yields a liquid weight of 0.07 ± 0.05 g, whereas speaking ten times produces a weight of 0.003 ± 0.001 g.

Size distributions of droplets from aerosols released when speaking or coughing were measured using laser diffraction employing Malvern Spraytech with a 300 mm lens. In this configuration, drop sizes between 0.2 *µ*m and 2 mm can be measured. Speaking and coughing is done directly into the laser beam, and data acquisition is done in the “fast acquisition” mode so that there is no dead time and the drop size distribution is measured before evaporation. For coughing, the volumetric distribution measured using laser diffraction shows that on average, 98% ± 1% of the volume of the spray is contained in the large drops (100 *μ*m–1000 *μ*m). For the small aerosol droplets, this amounts to ∼20 × 10^6^ microdroplets produced in a single cough and ∼7 × 10^6^ for speech. For COVID-19, thus from symptomatic patients, the viral RNA load in the undiluted oral fluid or sputum has been found to be 10^4^–10^6^ copies/ml.^25–28^ During infection, there are major changes in viral load, and the rate at which these changes happen could be related to the severity of the COVID-19 symptoms. While in some cases, very high viral loads up to ∼10^11^ copies/ml have been reported,^26^ a relation with the severity of the symptoms has not been firmly established so far. As such, following Ref. 25 to avoid underestimation, we used a number of 7 × 10^6^ copies/ml in respiratory samples in our primary analysis. The total number of virus particles present in the total volume of only the microdroplets is then 10^4^, implying that only one in 2000 aerosol droplets contains a virus particle.

### Persistence of aerosols

The persistence of these aerosol droplets in the air is of the greatest concern regarding community transmission of SARS-CoV-2 in public spaces. This airborne time is governed by evaporation and gravity-driven sedimentation toward the floor. The latter can be explained by balancing the forces of gravity (*F* = *mg*) and air drag (*F* = 6*πηRU*, with *η* being the air viscosity, *R* being the droplet radius, and *U* being the falling velocity), from which it follows that a droplet with a radius of 5 *μ*m will take 9 min to reach the ground from an initial height of 1.5 m. This time will even increase by the evaporation of the liquid phase of the droplet. Sputum droplets are known to consist for 1%–10% of their volume of solid solutes.^29^ Consequently, they will not evaporate completely but leave a “solid” core residue. For microdroplets smaller than 10 *µ*m in radius, the contraction to the solid core having half of the original droplet size (i.e., ∼10% of the initial volume) happens within a second in quiescent air with a relative humidity (RH) of 50%,^29^ and a droplet half the size stays airborne four times longer.

**FIG. 2.**
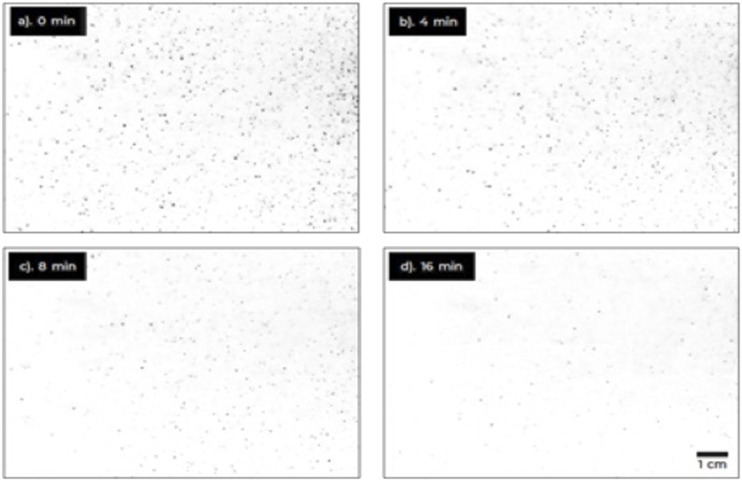
(a)-(d) Laser-illuminated aerosol droplets at different times after initial spraying. Initially (a), droplets have a maximum sedimentation velocity of about 2 cm/s, corresponding to droplets of about 25 *µ*m in diameter. In the 16 min frame (d), the fastest moving droplet has a sedimentation velocity of at most 1 mm/s, corresponding to a droplet of about 4 *µ*m–5 *µ*m in diameter.

A laser light sheet was used to track microdroplets similar to those produced by coughing and speaking. To mimic small respiratory droplets, droplets were generated with a Rayleigh jet nozzle chip (Medspray®) yielding the same droplet size distribution as droplets from a typical cough. To achieve this, we use a mixture of 1% glycerol and 99% ethanol; within a second, ethanol evaporates, yielding polydisperse non-evaporating droplets of glycerol with a median mass aerodynamic diameter (MMAD) of 5 ± 3 *µ*m, similar to the microdroplets produced by coughing or speaking. The number of drops passing through the laser sheet suspended in the center of our 2 × 2 × 2 m^3^ experimental chamber was analyzed by processing of the images using a home-built Python algorithm that detects the illuminations caused by the droplets. Typical results are shown in Fig. 2 and capture the reduction in the number of droplets over time due to coupled effects of sedimentation, horizontal displacement, and evaporation. The smallest air currents will make the aerosol concentration rather homogeneous. This was verified by measuring aerosol concentrations at different locations in the room.

If these aerosol droplets are a vector of transmission for the SARS-CoV-2 virus, how the number of droplets decreases as a function of time will have a significant influence on the potential airborne transmission of SARS-CoV-2. To predict the evolution in the number of microdroplets, the evaporation and sedimentation can be accounted for to calculate the number of airborne aerosol particles with knowledge of the initial droplet size distribution.

### A simple model for the persistence

The evaporation of a spherical droplet in an environment with a known relative humidity (RH) can be evaluated using the diffusion model presented and validated in Ref. 30. The rate of change in the mass of the droplet, *m*_*d*_(*t*), is given by

$$\frac{\partial m_{d}\left(t\right)}{\partial t}=4\pi R^{2}\left(t\right)D_{va}{\left.\frac{\partial C\left(r,t\right)}{\partial r}\right|}_{r=R\left(t\right)},$$ (1)

where *R*(*t*) is the radius of the droplet, *D*_*va*_ is the diffusivity of water vapor in air, and *C*(*r*, *t*) is the water vapor concentration along direction *r*. Assuming that the droplets are sufficiently spaced and that the relative humidity of the air in which they are falling through does not change, the final term can be written as

$${\left.\frac{\partial C\left(r,t\right)}{\partial r}\right|}_{r=R\left(t\right)}=\left(C\left(r=\infty \right)-C\left(\right.R\left(t\right),t\right)\left(\frac{1}{R\left(t\right)}+\frac{1}{\sqrt{\pi Dt}}\right).$$ (2)

The water vapor concentration at the surface of the droplet [i.e., *r* = *R*(*t*)] is given by the equilibrium vapor pressure, *ρ*_*vap*_, of the environment and, very far away from the droplet surface [i.e., *r* ≫ *R*(*t*)], is given by the product of the RH of the environment and *ρ*_*vap*_, resulting in

$$\frac{\partial m_{d}\left(t\right)}{\partial t}=4\pi R^{2}\left(t\right)D_{va}\rho_{vap}\left(RH-1\right)\left(\frac{1}{R\left(t\right)}+\frac{1}{\sqrt{\pi Dt}}\right).$$ (3)

Assuming that the solids (salt, proteins, and possibly virus particles) constitute a “spherical core” of the droplet, the mass, *m*_*d*_, of the droplet at any time is given by

$$m_{d}\left(t\right)=\frac{4\pi }{3}R_{0}^{3}\rho_{s}+\frac{4\pi }{3}\left(R^{3}\left(t\right)-R_{0}^{3}\right)\rho_{w},$$ (4)

where *ρ*_*s*_ is the density of the solid found in human mucus/saliva (i.e., 1500 kg/m^3^) from Ref. 31 and *ρ*_*w*_ is the density of liquid water. Differentiating Eq. (4) with respect to time and combining the result with Eq. (3) give a non-separable differential equation for the evolution in size of the droplet due to evaporation, where evaporation stops when the droplet is completely composed of the solid fraction or when *R*(*t*) = *R*_0_,

$$\frac{\partial R\left(t\right)}{\partial t}=\frac{\rho_{vap}D_{va}}{\rho_{w}}\left(RH-1\right)\left(\frac{1}{R\left(t\right)}+\frac{1}{\sqrt{\pi D_{va}t}}\right).$$ (5)

**FIG. 3.**
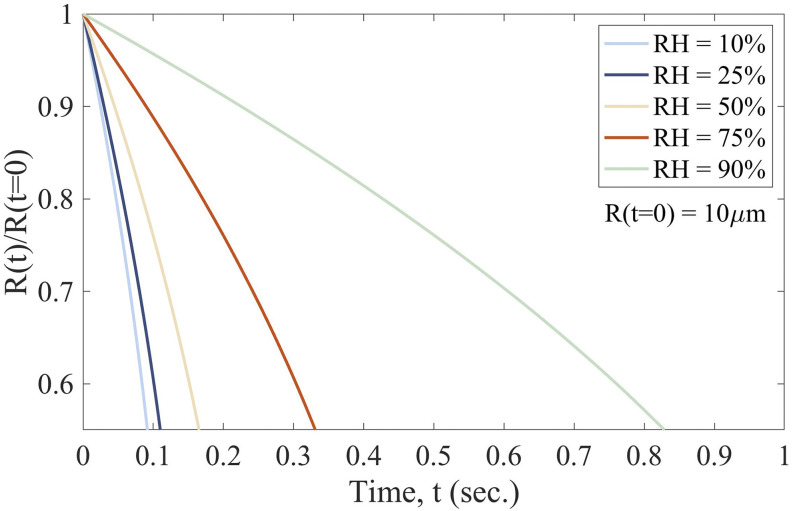
Influence of the relative humidity (RH) on the evaporation kinetics of a droplet with *R*(*t* = 0) = 10 *µ*m.

For the purpose of the following calculations, it is taken that the solid core *R*_0_ of each droplet is half of the initial size *R*(*t* = 0) and corresponds to an initial density of ∼1080 kg/m^3^ for the water–solute mixture. Figure 3 displays solutions to Eq. (5) for the largest micro-droplet sizes and shows the influence of the RH on the evaporation kinetics of a 10 *µ*m droplet. Within 1 s, the evaporation of the small micro-droplets is complete, resulting in a solid core.

Due to the fact that the evaporation occurs quickly, the dominant mode of decline in suspended droplets is sedimentation. As we will show below, the exponential decay in the number of drops that we observe can be quantitatively accounted for by taking only the sedimentation of already evaporated droplets into account. At all times, the droplets are assumed to be vertically falling at their terminal velocities described by Stokes flow,

$$\frac{\partial h\left(R\left(t\right),t\right)}{\partial t}=\frac{2\left(\rho_{d}\left(t\right)\right)}{9\eta }gR^{2}\left(t\right).$$ (6)

**FIG. 4.**
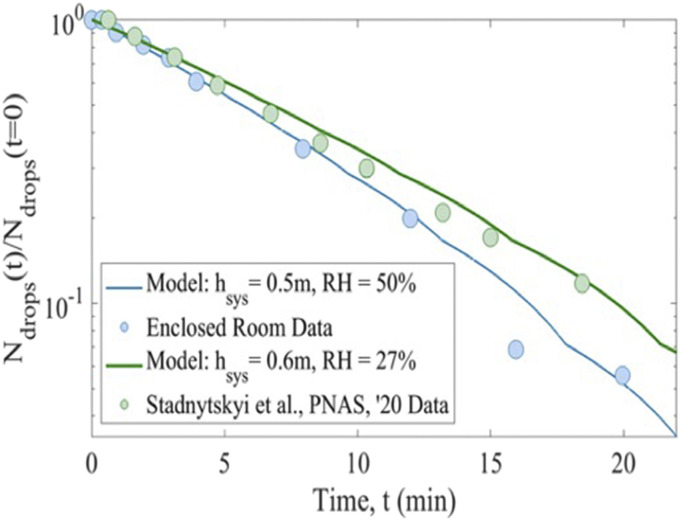
Normalized number of droplets as a function of time as determined experimentally (blue circles) compared to the data of Ref. 12 (green circles). Solid lines are model outcomes for both sets of data, with input parameters relative humidity (RH) and system height (*h*_*sys*_).

This describes the rate of change in the height, *h*(*R*(*t*), *t*), through which the droplet has fallen where *ρ*_*a*_ is the density of air and g is the acceleration due to gravity. By solving Eqs. (5) and (6) numerically, the progressive evaporation and sedimentation of the droplets are coupled and comparable to models presented in Refs. 5 and 32. For the framework presented herein, how the number of droplets in a given volume evolves can be predicted, allowing the persistence calculations in Fig. 4 to be made. For this calculation, it is assumed that the droplets of each size class have a uniform random initial height in the volume in which they progressively sediment. From the particle size distribution, the total number of particles of each size class, *N*(*R*(*t* = 0)), initially in the volume can be obtained. The evolution in the total number of particles for each size class is then directly given by $N\left(R\left(t\right),t\right)=N\left(R\left(t=0\right)\right)\frac{h\left(R\left(t\right),t\right)}{h_{sys}}$, taking *h*(*R*(*t*), *t*) to always be smaller than the system height *h*_*sys*_ in which dispersion experiments are made and the computational domain height in which the sedimentation and evaporation of the droplets are calculated. The total number of droplets in the system, *N*_*total*_, at any time *t* is then the discrete summation of this number over all particle sizes, *n*, for which *h*(*R*(*t*), *t*) < *h*_*sys*_.

Figure 4 shows that the derived system of equations and model system can directly predict the persistence of the aerosol particles with knowledge of the system size, initial size distribution of the aerosol droplets, and relative humidity. Explicit calculation shows that the half-life reduces nearly 50% when the relative humidity is 100%—corresponding to conditions in which there is no evaporation of aerosol. As expected, when no evaporation occurs, the droplets fall faster through the system due to their nominally larger size and higher terminal velocities. The decrease in the number of microdroplets in the system due to the effects of a higher relative humidity then implies a lower-likelihood of aerosol mediated transmission of CoV-2, which corresponds to other studies^33,34^ that show that higher relative, and absolute, humidity environments may lead to lower infectivity rates of influenza and other respiratory infections. Based on these results, a more general model can be derived to explain the exponential decline in droplets. Given a number *N*_*o*_ of drops with diameter *D*, and in view of the experimental results, it is reasonable to assume that the decrease in time will be exponential: $N\left(D,t\right)=N_{o}e^{-\alpha D^{2}t}$, with *α* being an empirical constant independent of the droplet diameter *D*. A good estimate is *α* ≅ *ρg*/18*ηh*, with *h* being a typical sedimentation height. The life time of a micro-droplet is then characterized by the exponent in Eq. (6), given by *t*_*life*_ ≡ 1/*αD*^2^.

In case of droplets with a varying size distribution, we collect the different droplet sizes and obtain $N_{total}\left(D,t\right)=\sum_{i=1}^{i=n}N_{i}e^{-\alpha D^{2}t}$.

Figure 4 compares our predictions for droplet persistence results with our own results and those reported by others.^12^ It shows that the model accurately captures the exponential decline in the number of droplets over time for both experiments and suggests the decline is, to a small extent, influenced by the evaporation of the droplets (i.e., the relative humidity of the environment) but dominated by the sedimentation. Additionally, from Fig. 4, it can be concluded that the time to half the original number of droplets in the system (i.e., the half-life) is between 5.5 min and 7 min. These lines are not fits but outcomes of the derived analytical model. The small discontinuities are a result of using, in the model, the original binned values of initial droplet sizes and discretely modeling the particles. Using continuous distributions for the initial droplet sizes systematically removes the discontinuities.

### Estimate of infection risk

**FIG. 5.**
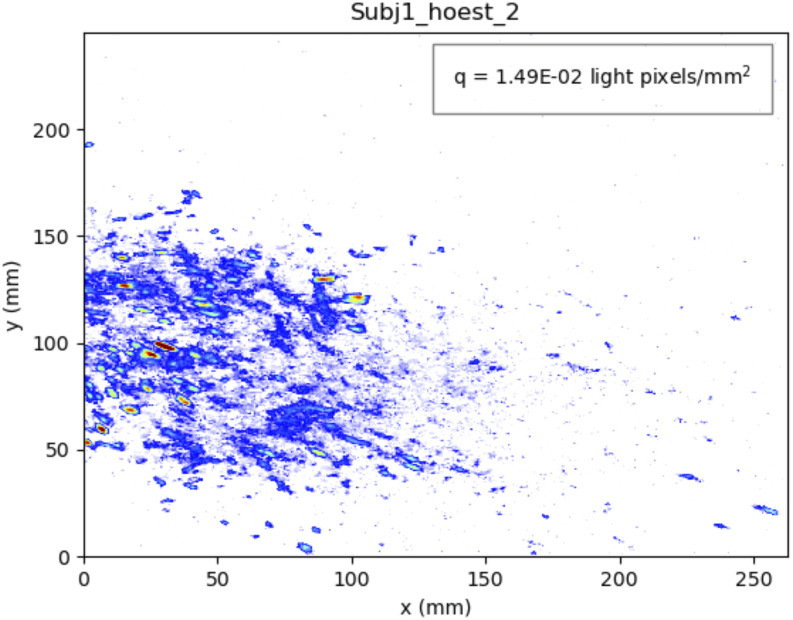
Picture and movie of the droplets produced by coughs of a high emitter. Multimedia view: https://doi.org/10.1063/5.0027844.110.1063/5.0027844.1

This then allows us to estimate how many virus particles one would inhale while inside a room where an infected person coughed a single time. The highest probability of infection occurs when a person enters a poorly ventilated and small space where a high emitter has just coughed and inhales virus-carrying droplets. We model coughing in our 2 × 2 × 2 m^3^ unventilated space that could represent, e.g., a restroom. The drop production by coughing was found to be very similar for six out of the seven emitters. We find peak values of 1.18 ± 0.09 × 10^3^ pixels that light up in the field of view of our laser sheet (21 × 31 cm^2^). This directly corresponds to the volume of emitted droplets;^12^ the high emitter produced 1.68 ± 0.20 × 10^4^ lit up pixels, more than an order of magnitude larger. Figure 5 (Multimedia view) shows the cough of the “superemitter.”

**FIG. 6.**
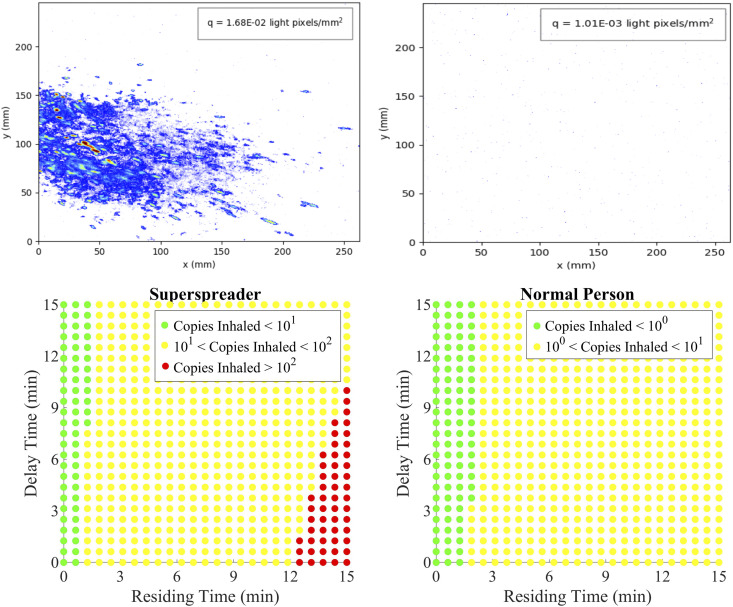
Instantaneous pictures of the droplets produced by coughs of a high emitter (a) and a normal emitter (b) as detected with laser sheet imaging. The cough volumes allow us to estimate the number of inhaled virus particles as a function of (i) the delay between the cough and a healthy person entering the room and (ii) the time the healthy person spends in the room [(c) and (d)].

Based on these numbers and the earlier measured volume and drop size, we can calculate the amount of virus inhaled by a person entering and staying in the same room where an infected person produced the droplets as a function of entrance delay and residence time. As detailed above, the calculation assumes a viral load of 7 × 10^6^ copies/ml of saliva.^25^ We also assume a single inhalation volume of 0.0005 m^3^ (tidal volume 6 ml/kg body weight for an adult man) and a normal respiratory rate of ∼16 inhalations/min.^35^ In Fig. 6, we compare the results for the high emitter with those for a regular (low) emitter on the basis of the amount of light scattered from droplets produced by a single cough.

The number of virus particles needed to infect a single individual, *N*_*inf*_, needs to be considered to translate these findings into risk of infection. This obviously also depends on factors such as the vulnerability/susceptibility of the host; in addition, as detailed in Ref. 36, the respiratory infectivity for SARS-CoV-2 is not yet well known. In the absence of data on SARS-CoV-2, the most reasonable assumption is that the critical number of virus particles to cause infection is comparable to that for other coronaviruses, including SARS-CoV-1, and influenza virus. In that case, *N*_*inf*_ ∼ 100–1000, which corresponds to ∼10 PFU to 100 PFU where PFU denotes plaque forming units, the standard way of expressing whether a virus is infectious or not.^36–38^ If we adopt a conservative approach and assume the upper limit of this range (*N*_*inf*_ ∼ 100), we find that our unventilated 2 × 2 × 2 m^3^ space contaminated by a single cough is relatively safe for residing times less than 12 min due to the low virus content of the aerosol particles. Additionally, the maximal number of inhaled viral copies by a person entering the room after the high emitter has coughed is ∼120 ± 60, where the error margin comes from variation in relative volume of small and large drops produced by a cough. If the infected person is a regular emitter, the probability of infecting the next visitor of the confined space by means of a single cough for any delay or residence time is therefore rather low. For speech, due to the low volumes emitted, this probability is even smaller. Nevertheless, prolonged speaking produces very large numbers of aerosols that could result in droplet accumulation to levels far higher than that in coughing or sneezing, thereby leading to an increased risk. Our small non-ventilated room can be looked upon as a “worst-case”: in better ventilated, large rooms, aerosols become diluted very rapidly.^13^ The methods described here do allow for a complete modeling of the probability of infection also for other types of rooms, with different particle inputs and ventilation characteristics. This should be a useful starting point for many hydrodynamic-based simulations of SARS-CoV-2 transmission that are currently being performed.^7,9,20^

## CONCLUSION

Our dynamic modeling of transmission of SARS-CoV-2 in confined spaces suggests that aerosol transmission is not a very efficient route, in particular from non-symptomatic or mildly symptomatic individuals that are likely to have low virus content in their saliva. Highly infected people having a large viral load in their saliva and superspreaders producing lots of aerosols are likely far more dangerous. Comparing aerosol transmission to other transmission routes, it is useful to realize that the large droplets that are believed to be responsible for direct and nosocomial infections may contain about 500 virus particles per droplet and are thus likely to also be very important in a mixed transmission model.

A limitation to our study is that we cannot easily take changes in virus viability inside microdroplets into account, which depend on the local microenvironment of the aerosol gas clouds as produced under different circumstances.^39^ However, viable SARS-CoV-2 in aerosols can be found after several hours,^40^ and as such, this limitation will not likely affect our main conclusion. Importantly, our results do not completely rule out aerosol transmission. It is likely that large numbers of aerosol droplets, produced by continuous coughing, speaking, singing, or certain types of aerosol-generating medical interventions, can still result in transmission, in particular in spaces with poor ventilation.^13^ Our model explains the rather low reproduction number of SARS-CoV-2 in environments where social distancing is practiced compared to the reproduction numbers of other “true” airborne pathogens.^22,41,42^ For a “true” airborne virus such as measles, the R-factor is 12–18, whereas the current best estimate for SARS-CoV-2 is about 2.5.^43^ This suggests that direct droplet transmission and fomite transmission are relatively more important ways of transmission than airborne transmission, for which R-values are generally (very) high. The calculation presented here allows us to do a risk estimation based on what we now know about the virus; in the case of new insights, the parameters of our model can be readily modified to incorporate these. The interpretation of the associated risk is necessarily subjective; what is acceptable as an infection probability is beyond the scope of this paper.

## DATA AVAILABILITY

The data that support the findings of this study are available from the corresponding author upon reasonable request. Part of this study included measurements from subjects, which were approved by a local ethical committee (AMC2020_098/NL73585.018.20).
